# Use of bisphosphonates in advanced prostate cancer: Current status

**DOI:** 10.4103/0970-1591.30268

**Published:** 2007

**Authors:** Wolfgang Lilleby

**Affiliations:** Radium Hospital, Oslo, Norway. E-mail: wolfgang.lilleby@medisin.uio.no

The main difference between the reviewed paper and my scientific point of view is the acceptance of bisphosphonate therapy (BT) in daily practice for patients with advanced prostate cancer. Bisphosphonate therapy has been shown to alleviate pain which certainly has an impact on patients' quality of life and skeleton-related events (SRE) at 15 months and two years follow-up (Saad F. JNCI, 2004). Reduction in fractures translates directly into diminished hospitalization, costs and improves the patients' well-being.

Contrary, the authors mean that the use of bisphosphonates is not established.

Nevertheless, taxan-based chemotherapy (docetaxel) is the first treatment shown to increase overall survival in patients with hormone-resistant prostate cancer and is today's standard treatment of patients in Western countries. Secondary hormone treatment and administration of bisphosphonates are other established alternatives for palliation of symptoms in patients with hormone-resistant prostate cancer. The existing data support this general statement, but use of bisphosphonates should be restricted to certain conditions such as recommended by the NUCG (guidelines from the Norwegian Urological Cancer Group): BT should be considered in patients with advanced prostate cancer as part of the palliative compassionate care treatment to reduce pain, SRE and use of opiates. Taxan-based chemotherapy should have priority without the combination of BT. The efficacy of combined treatment should be tested in trials such as the ongoing NEPRO study (docetaxel alone or in combination with risdronate). Patients with fractures should get BT every four weeks and orally calcium/vitamin D supplement.

